# Comparing lethal dose ratios using probit regression with arbitrary slopes

**DOI:** 10.1186/s40360-018-0250-1

**Published:** 2018-10-05

**Authors:** Chengfeng Lei, Xiulian Sun

**Affiliations:** Wuhan Institute of Virology, Chinese Academy of Sciences, Wuhan, 430071 Hubei China

**Keywords:** Toxicity, Probit regression, Lethal dose ratio, Maximum likelihood

## Abstract

**Background:**

Evaluating the toxicity or effectiveness of two or more toxicants in a specific population often requires specialized statistical software to calculate and compare median lethal doses (LD_50_s). Tests for equality of LD_50_s using probit regression with parallel slopes have been implemented in many software packages, while tests for cases of arbitrary slopes are not generally available.

**Methods:**

In this study, we established probit-log(dose) regression models and solved them by the maximum likelihood method using Microsoft Excel. The *z*- and *χ*^2^-tests were used to assess significance and goodness of fit to the probit regression models, respectively. We calculated the lethal doses (LDs) of the toxicants at different significance levels and their 95% confidence limits (CLs) based on an accurate estimation of log(LD) variances. We further calculated lethal dose ratios and their 95% CLs for two examples without assuming parallel slopes following the method described by Robertson, et al., 2017.

**Results:**

We selected representative toxicology datasets from the literature as case studies. For datasets without natural responses in the control group, the slopes, intercepts, *χ*^2^ statistics and LDs calculated using our method were identical to those calculated using Polo-Plus and SPSS software, and the 95% CLs of the lethal dose ratios between toxicants were close to those calculated using Polo-Plus. For datasets that included natural responses in the control group, our results were also close to those calculated using Polo-Plus and SPSS.

**Conclusion:**

This procedure yielded accurate estimates of lethal doses and 95% CLs at different significance levels as well as the lethal dose ratios and 95% CLs between two examples. The procedure could be used to assess differences in the toxicities of two examples without the assumption of parallelism between probit-log(dose) regression lines.

**Electronic supplementary material:**

The online version of this article (10.1186/s40360-018-0250-1) contains supplementary material, which is available to authorized users.

## Background

In toxicological, entomological and environmental studies, doses of toxicants that kill a defined proportion of organisms, e.g., the median lethal dose (LD_50_) which kills 50% of the population, are typically used as indicators of acute toxicity. Comparing the activities of different toxicants in a specific population or determining the relative susceptibilities of different populations to a single toxicant are common research goals. The relative potency, which assumes that the regression lines of the two toxicants being compared are parallel, provides a convenient comparison of the toxicities of two toxicants [1].

However, in practice, many regression lines are not parallel, particularly those derived from bioassays of toxicants with different modes of action, or from same-action toxicants administered to populations with different resistance levels. The 95% confidence limits (CLs) of a lethal dose ratio can be calculated by estimating the slopes and intercepts of two probit regression lines and constructing their variance and covariance matrices. The 95% CLs of this ratio indicate whether the lethal doses of the two toxicants are statistically different from one another [2]. Polo-Plus software, developed by Robertson et al. [3], separately analyzes the data for each substance using probit or logit models based on the joint probability of all observations and calculates lethal dose ratios and their CLs at different significance levels. IBM SPSS provides solution to calculate the lethal doses with 95% CLs based on probit or logit models, and also the relative median potency (RMP) assuming that the two regression lines are parallel [4].

In this study, we calculated lethal doses and 95% CLs of toxicants at different significance levels, as well as the lethal dose ratio and its 95% CLs for two toxicants, from probit-log(dose) regression models constructed using the maximum likelihood method in Microsoft Excel. The effectiveness of this method was compared with that of Polo-Plus and IBM SPSS.

## Methods

### Construction of probit-log(dose) regression models for a single toxicant or population

For a population treated with serial doses (*i*) of a toxicant, in which *n* subjects were treated and *r* subjects exhibited a characteristic response to each dose, the empirical proportion (p^*^) of responders was given by

1 $$ {p}_i^{\ast }=\frac{r_i}{n_i}. $$

where *i* = 1 to *k* and *k* indicated the number of toxicant doses.

If the characteristic response occurred in the control group (natural response) with proportion *C*, the proportions of responders were corrected using the Abbott equation for each treatment dose [5]:

2 $$ {p}_i=\frac{p_i^{\ast }-c}{1-c}. $$

The corrected proportion (*p*_*i*_) was then converted to a probit value (*y*_*i*_) [1]:

3 $$ {y}_i={\varPhi}^{-1}\left({p}_i\right), $$

which was calculated as *y*_*i*_ = NORM.S.INV(*p*_*i*_) in Excel.

A provisional regression line between *y*_*i*_ and the logarithm of the dose (*x*_*i*_) was established:

4 $$ {y}_i={\alpha}_0+{\beta}_0{x}_i. $$

In the regression equation, *i* = 1 to *m*, where *m* is the number of toxicant doses at which the corrected proportion was not equal to 1 or 0. The intercept (*α*_0_) and slope (*β*_0_) could be calculated by the least-squares procedure and were retrieved using the INTERCEPT(*y*_*i*_, *x*_*i*_) and SLOPE(*y*_*i*_, *x*_*i*_) functions, respectively, in Excel.

We then calculated the expected probits (*Y*) for all dose sets, included those where the corrected proportion was 1 or 0:

5 $$ {Y}_i={\alpha}_0+{\beta}_0{x}_i. $$

In Eq. (5), *i* = 1 to *k*.

We next calculated the expected response proportion (*P*_*i*_) for each dose set [1].

6 $$ {P}_i=\varPhi \left({Y}_i\right)\ast \left(1-C\right)+C, $$

where *Φ*(*Y*_*i*_) returned the cumulative probability of the standard normal distribution corresponding to (*Y*_*i*_), obtainable using the NORM.S.DIST (*Y*_*i*_) function in Excel, and *C* was the natural response proportion, if one existed, in Eq. (2).

The working probit (*y*_*i*_) was calculated from the following eq. [1]:

7 $$ {y}_i={Y}_i-\frac{P_i}{Z_i}+\frac{p_i}{Z_i}, $$

where

8 $$ {Z}_i=\frac{1}{\sqrt{2\pi }}{e}^{-0.5{Y}_i^2}. $$

An optimized set of expected probits was then derived from the linear regression equation of working probits weighted on *x*_*i*_, with each *y*_*i*_ being assigned a weight, *n*_*i*_*w*_*i*_, where *w*_*i*_ was the weighting coefficient. This was calculated as previously described [1]

9 $$ {w}_i=\frac{Z_i^2}{\left({P}_i+\frac{C}{1-C}\right)\left(1-{P}_i\right)}, $$

where *C* was the natural response proportion in Eq. (2).

The slope *β* of the working probit-log_10_(dose) regression equation was

10 $$ \beta =\frac{\sum_{i=1}^k{n}_i{w}_i\left({x}_i-\overline{x}\right)\left({y}_i-\overline{y}\right)}{\sum_{i=1}^k{n}_i{w}_i{\left({x}_i-\overline{x}\right)}^2}. $$

The intercept *α* of the working probit regression equation was

11 $$ \alpha =\overline{y}-\beta \overline{x}, $$

where *ȳ* was the average of *y* and $\overline{x}$ was the average of *x*:

12 $$ \overline{y}=\frac{\sum_{i=1}^k{n}_i{w}_i{y}_i}{\sum_{i=1}^k{n}_i{w}_i},\overline{x}=\frac{\sum_{i=1}^k{n}_i{w}_i{x}_i}{\sum_{i=1}^k{n}_i{w}_i}. $$

The standard error of *β* was [1]

13 $$ \sigma \left(\beta \right)=\sqrt{\frac{1}{\sum_{i=1}^k{n}_i{\left({x}_i-\overline{x}\right)}^2}}, $$

and the standard error of *α* was [6]

14 $$ \sigma \left(\alpha \right)=\sqrt{\frac{1}{\sum_{i=1}^k{n}_i{w}_i}+{\overline{x}}^2\sigma {\left(\beta \right)}^2}. $$

The χ^2^ statistic of the probit regression equation was [1]

15 $$ {X}^2={\sum}_{i=1}^k\frac{n_i{\left({p}_i-{P}_i\right)}^2}{P_i\left(1-{P}_i\right)}. $$

The significance level *p* of the χ^2^ statistic was calculated as the right-tailed probability of the chi-squared distribution (CHISQ.DIST.RT) with *k* – 2 degrees of freedom (*d.f.*).

A significant χ^2^ statistic (*p* < 0.05) might indicate either that the population did not respond independently or that the fitted probit-log(dose) regression line did not adequately describe the dose-response relationship in the test samples.

To get an optimal fit of the probit-log_10_(dose) regression, we substituted *α* and *β* for *α*_0_ and *β*_0_ and repeated the calculations of Eq. (5) to Eq. (15) until a stable χ^2^ appeared, indicating convergence. This procedure was a maximum likelihood (ML) method [1].

The significance of the slope was assessed using the *z* test [7],

16 $$ Z=\frac{\beta }{\sigma \left(\beta \right)} $$

If the absolute *z*-value was less than 1.96, the regression slope was not significant and the data were excluded from further analysis. Similarly, we might test the significance of the intercept (*α*).

The heterogeneity factor *h* of the regression equation was calculated to adjust for large χ^2^. *h* was defined as [1]

17 $$ h=\frac{\chi^2}{k-2}. $$

If *h* < 1, the model provided a good fit to the data. Otherwise, standardized residuals were plotted to identify outliers or other possible causes of poorness of fit [8]. Each residual defined the difference between the observed *r*_*i*_ and the expected response number (*n*_*i*_*P*_*i*_) for each dose. The residuals were standardized by dividing them by their standard errors, $\sqrt{n_i{P}_i\left(1-{P}_i\right)}$. For models providing a good fit, the standardized residuals fell mostly between −2 and 2 [8]. Standardized residuals distributed randomly showed no systematic patterns or tendencies toward positive or negative sign.

### Calculation of the lethal doses of toxicants or populations and their 95% CLs

In this step, we first calculated the logarithms of the doses (*θ*_π_) at which levels of interest (π) gave the expected response proportion:

18 $$ {\theta}_{\uppi}=\frac{y_{\uppi}-\alpha }{\beta } $$

where *y*_π_ was the π^th^ percentile of the probit distribution curve calculated in Excel using NORM.S.INV(π) for the probit distribution. For example, if π = 10, 50, 90 and 99, *y*_π_ was calculated as − 1.282, 0, 1.282 and 2.326.

The π^th^ lethal dose was then calculated as

19 $$ {\mathrm{LD}}_{\pi }={10}^{\uptheta_{\pi }}. $$

The standard error of *θ*_π_, *σ*(*θ*_*π*_), was given by [1]

20 $$ \sigma \left({\theta}_{\pi}\right)=\frac{1}{\beta}\sqrt{\frac{1}{\sum_{i=1}^k{n}_i{w}_i}+\frac{{\left({\theta}_{\pi }-\overline{x}\right)}^2}{\sum_{i=1}^k{n}_i{w}_i{\left({x}_i-\overline{x}\right)}^2}}. $$

The 95% CL of the LD_π_ was then given as

21 $$ {10}^{\theta_{\pi}\pm {t}_{0.05,k-2}\sigma \left({\theta}_{\pi}\right)}. $$

*t*_0.05, *k* − 2_ returned the two-tailed inverse of the Student’s *t*-distribution at *α* = 0.05 with *d.f. = k* - 2 [T.INV.2 T(0.05, *k*-2)].

The *g* value could be calculated to adjust if the confidence limits were valid. The *g* value was given as [9]:

22 $$ \mathrm{g}=\frac{t^2\sigma {\left(\beta \right)}^2}{\beta^2}{h}^{\ast }. $$

If *p* (χ^2^) was less than 0.15, *t* = 1.96 and h^*^ = 1; otherwise, h^*^ = *h* and *t* = *t*_0.05, *k* − 2_ [4]. If *g* exceeded 1, the CLs for the LD_π_ did not have practical importance [1].

The above steps were repeated to determine all parameters for the second toxicant for the same population, or the same toxicant in the second population.

### Comparison of lethal dose ratios of two toxicants or populations

If there were *l* toxicants or populations in the experiment, then we compared the LD_π_ values of the first (as a reference) to those of others. We first calculated the difference between the log(doses) yielding the expected response proportions (π^th^ percentile) for toxicants or populations 1 and *j* (*j =* 2 to *l*), *θ*_π1*j*_ = *θ*_π1_ - *θ*_π*j*_. Its standard error was given by [2]

23 $$ \upsigma \left({\theta}_{\pi 1j}\right)=\sqrt{\sigma {\left({\theta}_{\pi 1}\right)}^2+\sigma {\left({\theta}_{\uppi \mathrm{j}}\right)}^2}. $$

The ratio of the two lethal doses was then given as

24 $$ Ratio\left(1\mathrm{j}\right)={10}^{\theta_{\pi 1}-{\theta}_{\pi j}}, $$

and the 95% CLs were

25 $$ {10}^{\theta_{\pi 1j}\pm 1.96\sigma \left({\theta}_{\pi 1j}\right)}. $$

If the 95% CLs of this ratio excluded 1.0, the lethal doses of the two toxicants or populations were significantly different; otherwise, there was no evidence to reject the null hypothesis of equal LDs [2].

### Test for parallelism of the two regression equations

Although the above procedures did not assume equal slopes of the two regression lines, the specific LD_π_ level used depended on the parallelism of the regression lines. To examine parallelism of the two regression lines, we used the *z*-test [10]:

26 $$ z=\frac{\left|{\beta}_1-{\beta}_j\right|}{\sqrt{\sigma {\left({\beta}_1\right)}^2+\sigma {\left({\beta}_j\right)}^2}}. $$

If the absolute *z*-value exceeded 1.96, the two regressions were non-parallel; otherwise, they were parallel.

### Case studies

The above procedures might be executed on an Excel (version 2010 or higher) spreadsheet (provided as an Additional file 1). To compare the results of the ML procedure in Excel with those of Polo-Plus and SPSS, we extracted bioassay data from the literature: (1) chrysanthemum aphids dosed with Rotenone, Deguelin, and a mixture of these two substances [11], (2) three populations, Fairfax, Pixley and Schaefer, of the pest bug “Wicked Witch of the West” dosed with deltamethrin [12], and (3) two populations, BugRes and BugLab, of Godfather larvae dosed with pyrethroid [2] (Table 1).

**Table 1:** Selected bioassay data for toxicants in experimental populations

Toxicant [11]	*Dose*	*n* ^a^	*r* ^b^	Population [12]	*Dose*	*n*	*r*	Population [2]	*Dose*	*n*	*r*
Rotenone	2.6	50	6	Fairfax	0	30	0	BugRes	0	60	3
	3.8	48	16		2	48	12		3	60	9
	5.1	46	24		3	50	15		10	60	19
	7.7	49	42		5	50	31		20	60	32
	10.2	50	44		7	48	31		40	60	38
Deguelin	5.1	49	16		10	59	52		50	60	46
	10.0	48	18	Schaefer	0	60	0	BugLab	0	60	5
	20.4	48	34		2	60	15		0.03	30	7
	30.2	49	47		3	120	41		0.1	30	7
	40.7	50	47		5	60	39		0.3	30	6
	50.1	48	48		10	120	110		1	30	3
Mixture^c^	2.5	47	7		50	120	119		3	30	3
	5.1	46	22	Pixley	0	359	7		7	30	10
	10.0	46	27		10	70	22		10	60	32
	15.1	48	38		20	70	38		15	30	22
	20.4	46	43		30	50	38		20	30	30
	25.1	50	48		50	50	48				

^a^*n* was the total number of subjects administrated at each dose

^b^*r* was the number of subjects exhibited a characteristic response to each dose

^c^“Mixture” was a mixture of Rotenone and Deguelin at 1:1

## Results

### Slopes, intercepts and significance testing of probit-log(dose) models fitted to the example data

When we implemented the ML procedure to solve the probit-log(dose) equations for the three sample data in Excel, for the datasets in which there was no natural response (e.g., Rotenone, Deguelin, Mixture, Fairfax and Schaefer), the slope (*β*) and intercept (*α*) estimates of the converged probit-log(dose) regression were identical to those calculated using Polo-Plus and SPSS (with two methods, SPSS^1^ and SPSS^2^, to include the natural response proportion, *C*, by inputting the value of *C* and calculating it from the data, respectively) (Table 2). The standard errors of both *β* and *α*, calculated by Eq. (13) and Eq. (14), were close but not identical to those calculated using Polo-Plus and SPSS (Table 2). When the data sets included natural responses (e.g., Pixley, BugRes and BugLab), *β* and *α*, as well as their standard errors, were close to those produced by Polo-plus and SPSS. The results of our method and Polo-Plus were closer to those calculated using SPSS^1^ method than those calculated using SPSS^2^ method (Table 2, Bold items).

**Table 2:** Slopes, intercepts and results of significance testing for the example data fitted to the probit-log(dose) regression models using the ML procedure (Excel), Polo-Plus and SPSS

	Example	Estimates				Standard error (*σ*)				*z* ^b^			
		Excel	Polo-Plus	SPSS^1a^	SPSS^2a^	Excel	Polo-Plus	SPSS^1a^	SPSS^2a^	Excel	Polo-Plus	SPSS^1a^	SPSS^2a^
*β*	Rotenone	4.213	4.213	4.213	4.213	0.481	0.478	0.478	0.478	8.767	8.809	8.809	8.809
	Deguelin	2.633	2.633	2.633	2.633	0.279	0.279	0.279	0.279	9.434	9.421	9.421	9.421
	Mixture	2.533	2.533	2.533	2.533	0.269	0.272	0.272	0.272	9.400	9.320	9.320	9.320
	Fairfax	2.598	2.598	2.598	2.598	0.352	0.353	0.353	0.353	7.370	7.369	7.369	7.369
	Schaefer	2.812	2.812	2.812	2.812	0.281	0.273	0.273	0.273	9.999	10.282	10.282	10.282
	**Pixley** ^c^	**2.982**	**2.917**	**2.915**	**4.897**	**0.401**	**0.402**	**0.401**	**1.200**	**9.999**	**7.248**	**7.264**	**4.080**
	**BugRes**	**1.730**	**1.551**	**1.545**	**1.703**	**0.270**	**0.252**	**0.229**	**0.532**	**6.402**	**6.148**	**6.736**	**3.202**
	**BugLab**	**5.541**	**5.461**	**4.941**	**3.631**	**0.960**	**1.062**	**0.948**	**0.716**	**5.771**	**5.142**	**5.215**	**5.071**
*α*	Rotenone	−2.887	−2.887	−2.887	−2.887	0.351	0.350	0.350	0.350	−8.225	−8.247	−8.247	−8.247
	Deguelin	−2.622	−2.622	−2.622	−2.622	0.342	0.339	0.339	0.339	−7.670	−7.743	−7.743	−7.743
	Mixture	−2.036	− 2.036	− 2.036	− 2.036	0.271	0.272	0.272	0.272	−7.519	−7.491	−7.491	−7.491
	Fairfax	−1.603	−1.603	−1.603	−1.603	0.250	0.249	0.249	0.249	−6.413	−6.435	−6.435	−6.435
	Schaefer	−1.622	−1.622	−1.622	−1.622	0.190	0.186	0.186	0.186	−8.530	−8.728	−8.728	− 8.728
	**Pixley** ^c^	**−3.666**	**−3.556**	**−3.552**	**−6.778**	**0.531**	**0.529**	**0.527**	**1.832**	**−6.903**	**−6.719**	**−6.741**	**−3.699**
	**BugRes**	**−2.387**	**−2.064**	**−2.053**	**− 2.338**	**0.384**	**0.367**	**0.315**	**0.908**	**−6.218**	**−5.618**	**− 6.512**	**−2.575**
	**BugLab**	**−5.690**	**−5.587**	**−4.935**	**−3.640**	**1.028**	**1.141**	**0.997**	**0.754**	**−5.535**	**−4.897**	**−4.951**	**−4.826**

^a^SPSS includes the natural responses proportion (*C*) by two methods: 1, inputting the value of *C*; and 2, calculating the corrected *p* from the data. The *d.f.* = *k* − 2 in method 1, while it was *k*-3 in method 2

^b^Polo-Plus used the *t*-ratio to test the significance of the linear regression. The significance criterion for the *t*-ratio (*α* = 0.05) was 1.96 (*t*-distribution with *d.f.* = ∞). This significance level was identical to that of the *z* test

^c^Bold items indicated the data sets included natural responses

The probit-log(dose) regression model assumes a linear relationship between the logarithm of serial doses and the probit of the response proportions. When *z-*tests (this study and SPSS) or the *t*-ratios (Polo-Plus) were used to evaluate the significance of the regressions, all *z* values and *t*-ratios for both *β* and *α* estimates calculated using all four methods exceeded 1.96 (Table 2), indicating that all regression parameters were significant. If the *z*-value for the slope was less than 1.96, the regression model would be insignificant and the dataset should be excluded from further analysis.

### Goodness-of-fits of the probit-log(dose) regressions

While *z*-tests evaluated whether a linear relationship existed between the probits and the log(dose), *χ*^2^ tests are usually used to test the goodness-of-fit of the log(dose)-probit regression model. For datasets that did not include natural responses, the *χ*^2^ and *h* values calculated in this study were identical to those calculated using Polo-Plus and SPSS (Table 3). When the datasets included natural responses, the *χ*^2^ and *h* values were close to those produced by Polo-plus and SPSS. Again, the results of our method and Polo-Plus were closer to those calculated using SPSS^1^ method than those calculated using SPSS^2^ method (Table 3, Bold items).

**Table 3:** Goodness-of-fit of the probit-log(dose) regression models calculated from the example data using the ML procedure (Excel), Polo-Plus and SPSS

Examples	*χ* ^2^				*h* ^b^				*g* ^c^
	Excel	Polo-Plus	SPSS^1a^	SPSS^2a^	Excel	Polo-Plus	SPSS^1a^	SPSS^2a^	Excel
Rotenone	1.729	1.729	1.729	1.729	0.576	0.576	0.576	0.576	0.050
Deguelin	12.026^d^	12.026^d^	12.026^d^	12.026^d^	3.006	3.006	3.006	3.006	0.260
Mixture	4.995	4.995	4.995	4.995	1.249	1.249	1.249	1.249	0.043
Fairfax	3.754	3.754	3.754	3.754	1.251	1.251	1.251	1.251	0.071
Schaefer	11.384^d^	11.384^d^	11.384^d^	11.384^d^	3.795	3.795	3.795	3.795	0.384
**Pixley** ^e^	**2.671**	**2.708**	**2.712**	**0.064**	**1.335**	**1.354**	**1.356**	**0.032**	**0.069**
**BugRes**	**1.382**	**1.358**	**1.362**	**1.266**	**0.461**	**0.453**	**0.454**	**0.633**	**0.094**
**BugLab**	**13.555**	**11.081**	**27.454**	**10.181**	**1.936**	**1.583**	**3.922**	**1.697**	**0.325**

^a^SPSS includes the natural responses proportion by inputting the value of *C*, and SPSS calculates the corrected *p* from the data

^b^*h*, heterogeneity factor (see Eq.(17)). SPSS did not give *h*. To compare the results from this study and Polo-Plus, it was shown as *h* = *χ*^2^/d.f. here

^c^The g value was calculated as Eq.(22). Polo-Plus and SPSS did not calculate the *g* values

^d^*χ*^2^ indicated the goodness-of-fit test was significant at *α* = 0.05

^e^Bold items indicated the data sets included natural responses

**Fig. 1:**
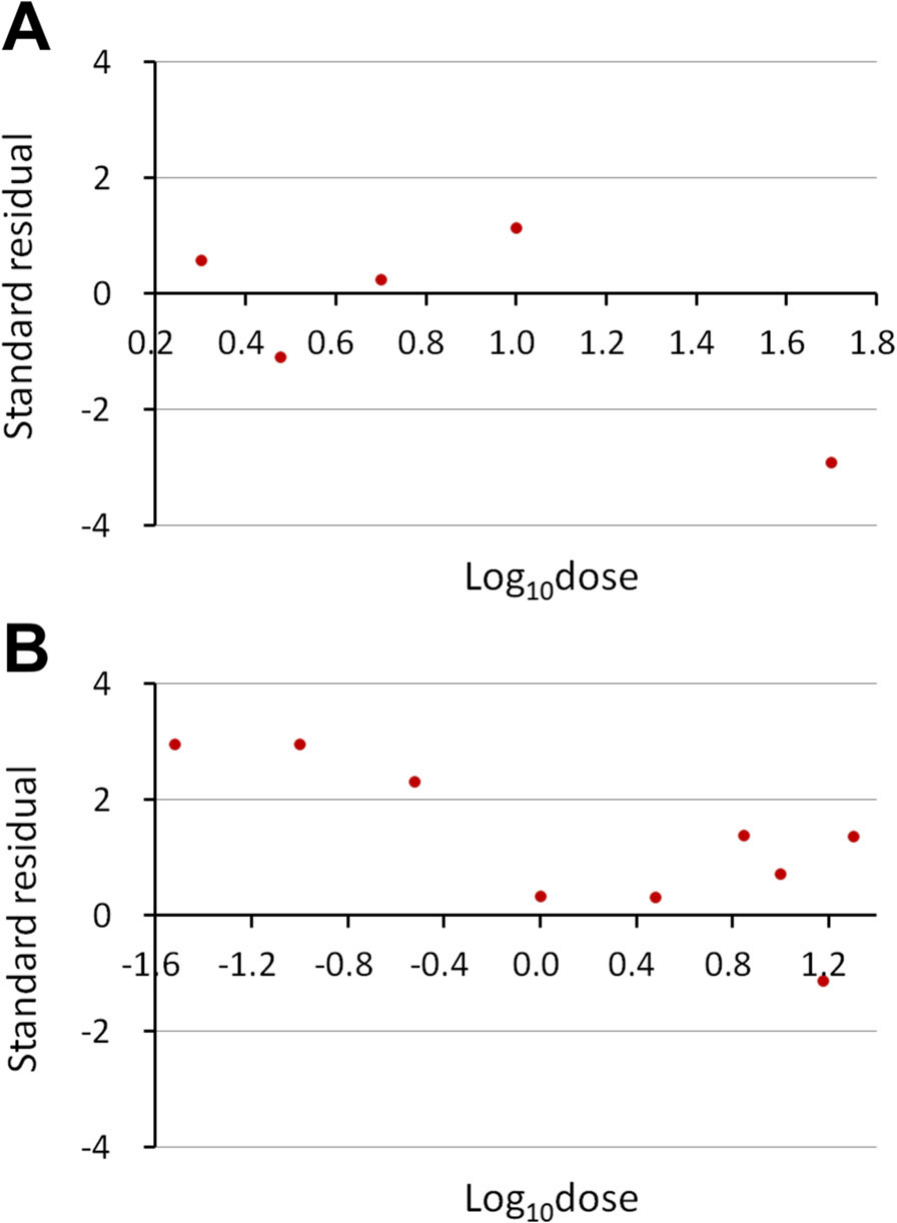
Standardized residuals versus log(doses) after fitting the Schaefer (**a**) and Buglab (**b**) dataset to probit-log(dose) models

For some datasets, *χ*^2^ was not significant but *h* was greater than 1 (Table 3). When standardized residuals were plotted against log(doses), one or more outliers were observed (outside the bounds of − 2 to 2) in the Schaefer and BugLab data. For the BugLab data especially, the standardized residuals were not distributed randomly and showed a tendency toward positive sign (Fig. 1), indicating that this data should be fitted using other models [13].

### LD_10_, LD_50_, LD_90_ and LD_99_ estimates with 95% CLs

We further compared the LD_π_s and their 95% CLs calculated using these four methods. For datasets that did not include natural responses, the LD_π_s calculated in this study were identical to those calculated using Polo-Plus and SPSS, and the 95% CLs of LD_π_s calculated using our method were close but not identical to those produced by Polo-Plus and SPSS (Table 4). For datasets that included natural responses, the LD_π_s and their 95% CLs were close but not identical to those calculated using Polo-plus and SPSS. The results of our method and Polo-plus were closer to those calculated using SPSS^1^ method than those calculated using SPSS^2^ method (Table 4, Bold items).

**Table 4:** LD_10_, LD_50_, LD_90_ and LD_99_ values with their 95% CLs for the example data fitted to probit-log(dose) regression models using the ML procedure (Excel), Polo-Plus and SPSS

Interested levels (π)	Samples	LD_π_ (95% CLs)			
		Excel	Polo-Plus	SPSS^1a^	SPSS^2a^
10	Rotenone	2.405 (1.756, 3.295)	2.405 (1.889, 2.833)	2.405 (1.889, 2.833)	2.405 (1.889, 2.833)
	Deguelin	3.229 (1.945, 5.360)	3.229 (0.606, 5.915)	3.229 (0.606, 5.915)	3.229 (0.606, 5.915)
	Mixture	1.986 (1.209, 3.263)	1.986 *(0.889, 3.059)*^b^	1.986 *(1.286, 2.672)*	1.986 *(1.286, 2.672)*
	Fairfax	1.329 (0.736, 2.400)	1.329 *(0.392, 2.112)*	1.329 *(0.820, 1.782)*	1.329 *(0.820, 1.782)*
	Schaefer	1.321 (0.872, 2.001)	1.321 (0.207, 2.247)	1.321 (0.207, 2.247)	1.321 (0.207, 2.247)
	**Pixley** ^c^	**6.307 (3.011, 13.210)**	**6.022 (0.393, 10.588)**	**6.011 (3.765, 7.969)**	**13.252 (5.512, 18.430)**
	**BugRes**	**4.355 (1.721, 11.023)**	**3.194 (1.143, 5.583)**	**3.157 (1.373, 5.105)**	**4.174 (0.082, 11.078)**
	**BugLab**	**6.246 (4.714, 8.275)**	**6.145 (2.450, 8.105)**	**5.488 (0.011, 8.109)**	**4.461 (0.927, 6.696)**
50	Rotenone	4.845 (4.122, 5.696)	4.845(4.363, 5.354)	4.846 (4.363, 5.354)	4.846 (4.363, 5.354)
	Deguelin	9.905 (7.658, 12.812)	9.905 (5.090, 14.626)	9.905 (5.090, 14.626)	9.905 (5.090, 14.626)
	Mixture	6.366 (4.981, 8.135)	6.366 *(4.564, 8.187)*	6.366 *(5.254, 7.484)*	6.366 *(5.254, 7.484)*
	Fairfax	4.139 (3.240, 5.288)	4.139 *(2.926, 5.482)*	4.139 *(3.511, 4.800)*	4.139 *(3.511, 4.800)*
	Schaefer	3.773 (3.110, 4.579)	3.773 (2.198, 5.717)	3.773 (2.198, 5.717)	3.773 (2.198, 5.717)
	**Pixley** ^c^	**16.967 (12.284, 23.436)**	**16.559 (8.096,24.636)**	**16.544 (13.963, 19.082)**	**24.208 (16.712, 29.114)**
	**BugRes**	**23.981 (16.593, 34.658)**	**21.413 (11. 546, 28.362)**	**21.318 (16.502, 27.590)**	**23.612 (6.574, 35.519)**
	**BugLab**	**10.638 (9.336, 12.121)**	**10.548 (7.912, 12.738)**	**9.971 (2.962, 14.238)**	**10.054 (6.699, 13.602)**
90	Rotenone	9.761 (7.323, 13.011)	9.761(8.405, 12.134)	9.762 (8.405, 12.134)	9.762 (8.405, 12.134)
	Deguelin	30.381 (22.388, 41.228)	30.381 (19.950, 77.517)	30.381 (19.950, 77.517)	30.381 (19.950, 77.517)
	Mixture	20.407 (14.636, 28.454)	20.407 *(15.015, 34.190)*	20.407 *(16.596, 27.120)*	20.407 *(16.596, 27.120)*
	Fairfax	12.892 (7.803, 21.299)	12.892 *(8.611, 36.089)*	12.892 *(10.006, 19.424)*	12.892 *(10.006, 19.424)*
	Schaefer	10.777 (7.559, 15.365)	10.777 (6.747, 50.379)	10.777 (6.747, 50.379)	10.777 (6.747, 50.379)
	**Pixley** ^c^	**45.645 (25.980, 80.196)**	**45.538 (28.964, 329.883)**	**45.533 (36.541, 64.751)**	**44.222 (36.854, 63.231)**
	**BugRes**	**132.040 (52.601, 331.448)**	**143.532 (88.364, 344.840)**	**143.975 (88.678, 333.43)**	**133.577 (82.497, 723.399)**
	**BugLab**	**18.118 (14.484, 22.665)**	**18.108 (14.508, 35.264)**	**18.118 (13.196, 1530.98)**	**22.662 (15.855, 84.406)**
99	Rotenone	17.278 (10.761, 27.743)	17.278(13.588,24.958)	17.278 (13.588, 24.958)	9.762 (8.405, 12.134)
	Deguelin	75.759 (44.790, 128.141)	75.759 (39.827, 460.545)	75.759 (39.827, 460.545)	75.759 (39.827, 460.545)
	Mixture	52.753 (29.785, 93.433)	52.753 *(32.074, 135.526)*	52.753 *(37.441, 87.710)*	52.753 *(37.441, 87.710)*
	Fairfax	32.548 (13.574, 78.046)	32.548 *(16.589, 209.890)*	32.548 *(21.149, 67.448)*	32.548 *(21.149, 67.448)*
	Schaefer	25.356 (13.882, 46.314)	25.356 (12.119, 412.504)	25.356 (12.119, 412.504)	25.356 (12.119, 412.504)
	**Pixley** ^c^	**102.28 (38.072, 274.763)**	**103.882 (49.732,4503.346)**	**103.939 (71.350,196.711)**	**72.273 (53.911,155.013)**
	**BugRes**	**530.489 (109.45, 2571.23)**	**676.988 (295.27, 3261.06)**	**683.244 (302.10, 2931.66)**	**548.646 (209.66, 26,126.13)**
	**BugLab**	**27.97 (19.047, 41.067)**	**28.133 (19.726, 97.529)**	**29.481 (17.762, 174,201.0)**	**43.958 (24.635, 485.621)**

^a^SPSS includes the natural responses proportion by inputting the value of *C*, and SPSS calculates the corrected *p* from the data

^b^Data in italic brackets indicated that he 95% CLs of LDπ calculated using Polo-Plus were not identical to those calculated using SPSS

^c^Bold items indicated the data sets included natural responses

### Comparison of lethal dose ratios between two samples

For datasets that did not include natural responses, the LD_π_ ratios calculated using our method were identical to those calculated using Polo-Plus and their 95% CLs were also close. For datasets that included natural responses, LD_π_ ratios and their 95% CLs calculated using our method were similar to those calculated using Polo-Plus (Table 5, Bold items). The LD_50_ ratios and their 95% CLs calculated using our method were closer to those calculated using Polo-Plus than to the relative median potency (RMP) calculated using SPSS (Table 5).

**Table 5:** Lethal dose ratios for the examples fitted to the probit-log(dose) regression models calculated by the ML procedure (Excel), Polo-Plus and SPSS

Interested levels (π)	Comparison	Lethal ratio (95%CL)		RMP (95%CL)^a^
		Excel	Polo-Plus	SPSS^2b^
10	Rotenone/Deguelin	0.745 (0.496, 1.119)	0.745 (0.494, 1.122)	
	Rotenone/Mixture	1.211 (0.812, 1.808)	1.211 (0.805, 1.824)	
	Fairfax*/*Scheafer	1.006 (0.645, 1.569)	1.006 (0.642, 1.577)	
	**Fairfax** ***/*** **Pixley** ^c^	**0.211 (0.128, 0.346)**	**0.221 (0.132, 0.369)**	
	**BugRes** ***/*** **BugLab**	**0.697 (0.376, 1.293)**	**0.520 (0.238, 1.138)**	
50	Rotenone/Deguelin	0.489 (0.398, 0.602)	0.489 (0.397, 0.603)	0.455 (0.173, 0.793)
	Rotenone/Mixture	0.761 (0.623, 0.929)	0.761 (0.621, 0.933)	0.710 (0.440, 1.005)
	Fairfax*/*Scheafer	1.097 (0.905, 1.329)	1.097 (0.902, 1.335)	1.106 (0.811, 1.550)
	**Fairfax** ***/*** **Pixley** ^c^	**0.244 (0.198, 0.301)**	**0.250 (0.201, 0.311)**	**0.261 (0.045, 0.571)**
	**BugRes** ***/*** **BugLab**	**2.254 (1.753, 2.898)**	**2.030 (1.478, 2.787)**	**3.898 (0.455, 4701.677)**
90	Rotenone/Deguelin	0.321 (0.243, 0.425)	0.321 (0.241, 0.428)	
	Rotenone/Mixture	0.478 (0.357, 0.642)	0.478 (0.354, 0.646)	
	Fairfax*/*Scheafer	1.196 (0.819, 1.747)	1.196 (0.814, 1.758)	
	**Fairfax** ***/*** **Pixley** ^c^	**0.282 (0.189, 0.422)**	**0.283 (0.186, 0.430)**	
	**BugRes** ***/*** **BugLab**	**7.288 (4.014, 13.232)**	**7.926 (4.077, 15.412)**	
99	Rotenone/Deguelin	0.228 (0.142, 0.366)	0.228 (0.140, 0.371)	
	Rotenone/Mixture	0.328 (0.199, 0.539)	0.328 (0.197, 0.546)	
	Fairfax*/*Scheafer	1.284 (0.667, 2.469)	1.284 (0.661, 2.493)	
	**Fairfax** ***/*** **Pixley**	**0.318 (0.158, 0.642)**	**0.313 (0.151, 0.651)**	
	**BugRes** ***/*** **BugLab**	**18.968 (6.820, 52.753)**	**24.064 (7.520, 77.007)**	

^a^RMP, relative median potency. We did not show the RMP of SPSS by inputting *C* methods because of different *C* values in the two samples

^b^SPSS calculates the corrected *p* from the data. We did not calculate RMP with SPSS by inputting *C* methods because of different *C* values in the two samples

^c^Bold items indicated the data sets included natural responses in the control group

When judged by whether the 95% CLs of lethal ratios included 1.0, all methods reached the same conclusions for toxicity differences between two samples (Table 5).

### Comparisons of two regression slopes

Parallelism between paired regression equations was examined using *z*-tests. The conclusions of our method for the five regression pairs were identical to those arrived at by Polo-Plus and SPSS, which used *χ*^2^ tests (Table 6).

**Table 6:** Tests of parallelism between the probit-log(dose) regression lines calculated using the ML procedure (Excel), Polo-Plus and SPSS

Comparison	Excel		Polo-Plus		SPSS^2a^	
	*z*	*Parallelism*	*χ* ^2^_( *d.f.* = 1)_	*Parallelism*	*χ* ^2^_( *d.f.* = 1)_	*Parallelism*
Rotenone vs Deguelin	2.844	Rejected	8.41	Rejected	10.216	Rejected
Rotenone vs Mixture	3.049	Rejected	9.68	Rejected	9.284	Rejected
Fairfax vs Scheafer	0.475	Accepted	0.23	Accepted	0.000	Accepted
Fairfax vs Pixley	0.720	Accepted	0.36	Accepted	0.598	Accepted
BugRes vs BugLab	3.821	Rejected	22.10	Rejected	24.840	Rejected

^a^We did not compare parallelism among the regression lines calculated by SPSS by inputting *C* methods because of different *C* values in the two samples

## Discussion

Many methods have been developed to calculate the lethal or effective doses of toxicants and their confidence limits. Probit analysis, developed by Bliss [14] and improved by Finney [11], is one such commonly-used method. To calculate the parameters of the probit-log(dose) regression, Finney suggested fitting the regression line by eye as precisely as possible and obtaining parameters, such as slopes and intercepts, of the provisional regression line at the first stage. Thereafter, one calculates the working probits *Y*, and repeats this process with the new set of *Y* values; when the iterations converge, this gives a precise estimate of the linear regression parameters [1]. In this study, we calculated slopes and intercepts for the provisional regression line by the least-squares procedure, and calculated working probits and performed the iteration procedure (ML) using the popular software program, Microsoft Excel. We obtained similar results to those obtained using Polo-Plus and SPSS.

Several software packages, such as Polo-Plus and SPSS, might be used to calculate the lethal doses and 95% CLs at different significance levels, and even test the equality of the lethal doses. Such professional statistical programs are difficult to handle for common toxicologists and environmental ecologists, and are easily abused. Excel in the Microsoft Office Package is the most popular statistical program around the globe. As to the Excel spreadsheet developed in this study, the users are easily to trace the procedure which is used to solve the regression equations, and calculate the CLs of a lethal dose and also the lethal dose ratios. They may further redevelop it easily according to their request.

*χ*^2^ values were used as indicators of the goodness-of-fit of the probit-log(dose) regressions as the iteration proceeded. The equations

27 $$ {\upchi}^2=\sum nw{\left(y-\overline{y}\right)}^2-\frac{{\left(\sum nw\left(x-\overline{x}\right)\left(y-\overline{y}\right)\right)}^2}{\sum nw\left(x-\overline{x}\right)} $$

or

28 $$ {\upchi}^2=\sum \frac{{\left(r- nP\right)}^2}{nP\left(1-P\right)} $$

could also be applied [1]. When there were no natural responses in the datasets, these two equations, along with Eq. (15), gave the same results when the iterations converged, and these results were identical to those produced by Polo-Plus and SPSS. When the datasets included natural responses, Eq. (27) always gave the smallest *χ*^2^ value, Eq. (28) always gave the largest value, while Eq. (15) gave an intermediate value which was closer to the output of Polo-Plus and SPSS (data not shown). During iteration for some datasets, the *χ*^2^ values produced from all these three equations might increase [1]. Most regression models converged after several iterations, and we reported the results after 20 iterations, as this was the default maximum used by SPSS.

Strictly speaking, the 95% CLs of LD_π_ were the values of *x* for which the boundaries of the fiducial band attained the relevant value of *y*_*π*_. The exact CLs of *θ*_*π*_ could be calculated by constructing the variance matrices of the slope (*var*(*β*)) and intercept (*var*(*α*)) and their covariance (*cov*(*α*,*β*)) matrices as follow [1, 9]:

29 $$ {\theta}_{\pi }+\frac{g}{1-g}\left({\theta}_{\pi }-\frac{\mathit{\operatorname{cov}}\left(\alpha, \beta \right)}{\mathit{\operatorname{var}}\left(\beta \right)}\right)\pm \frac{t}{\beta \left(1-g\right)}\sqrt{\mathit{\operatorname{var}}\left(\alpha \right)-2{\theta}_{\pi}\mathit{\operatorname{cov}}\left(\alpha, \beta \right)+{\theta_{\pi}}^2\mathit{\operatorname{var}}\left(\beta \right)-g\left(\mathit{\operatorname{var}}\left(\alpha \right)-\frac{\mathit{\operatorname{cov}}{\left(\alpha, \beta \right)}^2}{\mathit{\operatorname{var}}\left(\beta \right)}\right)}. $$

It has been theorized that, in practice, the method for determining 95% CLs of LD_π_ most often performed sufficiently good based on a trustworthy value for the variance of *θ*_π_ as Eq. (20) [1, 15]. It was suggested that 95% CLs of LD_π_ could be calculated using the formula ${10}^{\theta_{\pi}\pm 1.96\sigma \left({\theta}_{\pi}\right)}$ [15]. The results of this equation were close to those calculated using Eq. (29) when the dose number (*k*) was large (e.g., close to 10), while the CLs were much narrow than those calculated exactly using Eq. (29) when *k* was small. By contrast, the results given by Eq. (21) were nearer to those calculated exactly at different levels of *k*. The 95% CLs of LD_π_ calculated using Polo-Plus were often identical to those calculated using SPSS when there was no natural response, with some exceptions (e.g., the Mixture and Fairfax data; Table 4, italic brackets, although the *g* values were not large for both of these cases).

While it is common to find estimates of LDs obtained from probit analyses in the toxicology literature, it is less common to find a hypothesis test procedure to determine whether estimated differences between LDs are statistically significant [16]. Relative potency has been frequently used [1, 4], but this method assumes the regression lines being compared are parallel. When the regression lines were parallel, the LDs and their 95% CLs for two toxicants calculated from the two datasets simultaneously were similar to those calculated from the datasets separately. However, when the regression lines were not parallel, the LDs and their 95% CLs calculated from the two datasets simultaneously were quite different from those calculated from the datasets separately.

In cases where the data are suggestive of a trend toward significant differences between LD_50_s, the use of non-overlapping CLs for LD_50_ values has frequently been proposed as a criterion for assessing significance, while use of this criterion is thought to be conservative [17, 18]. An alternative method involves calculating the variances of *θ*_π_ using the delta-method:

30 $$ \mathit{\operatorname{var}}\left({\theta}_{\pi}\right)=\frac{1}{\beta^2}\left[\mathit{\operatorname{var}}\left(\alpha \right)+2{\theta}_{\pi}\mathit{\operatorname{cov}}\left(\alpha, \beta \right)+{\theta_{\pi}}^2\mathit{\operatorname{var}}\left(\beta \right)\right], $$

calculating the ratio of the LDs as in Eq. (24), then calculating the 95% CLs of the ratio as in Eq. (25) [2]. If the 95% CLs of the ratio include 1.0, the LDs of the two samples are not significantly different. We followed this procedure in this study, but we calculated the standard error of *θ*_π_ as in Eq.(20) by the maximum likelihood procedure. We obtained 95% CLs of the LD ratio similar to those obtained using Polo-Plus.

**Fig. 2:**
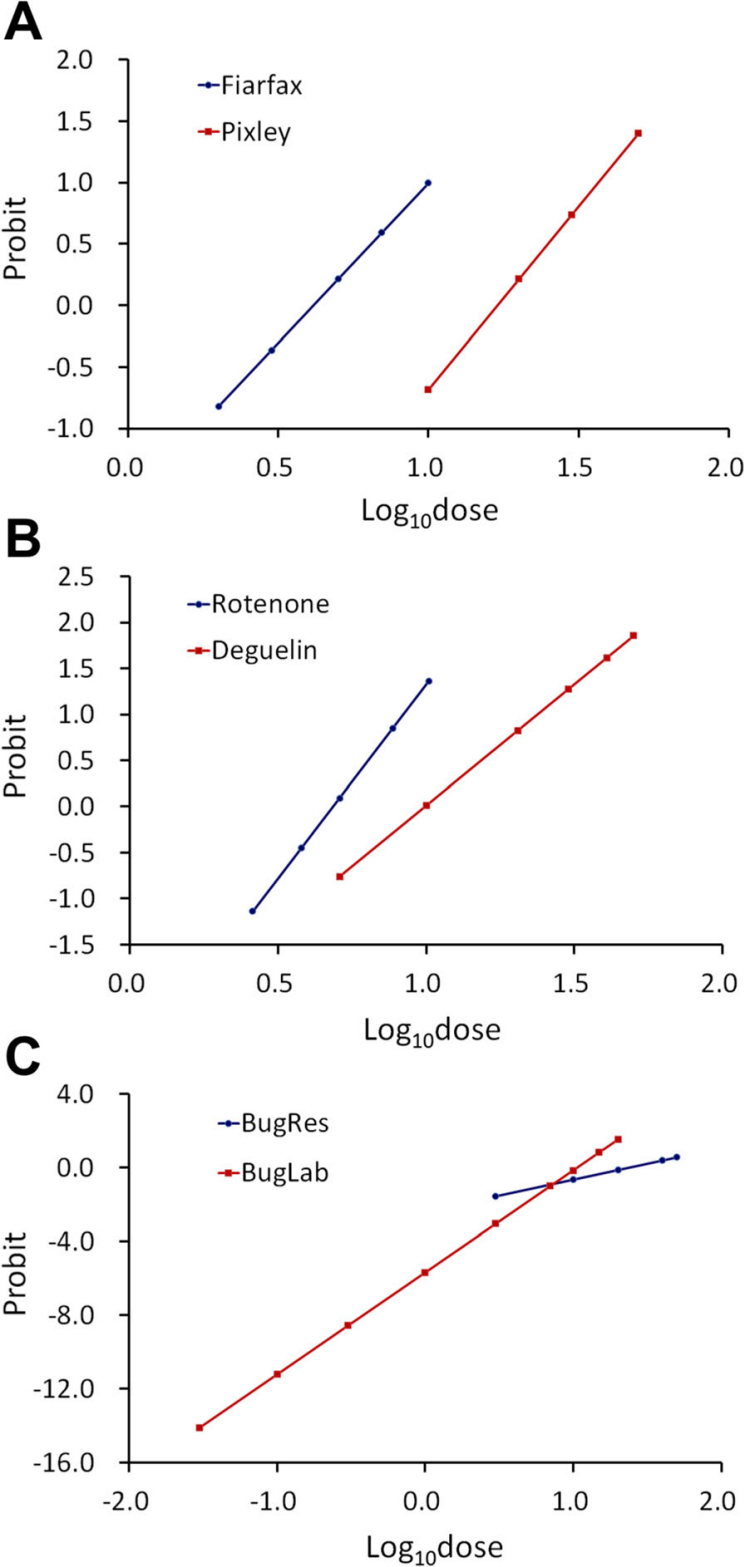
The three categories of parallelism between two regression lines. (**a**) Fairfax vs Pixley; (**b**) Rotenone vs Deguelin; (**c**) BugRes vs BugLab

Biologically, the slope of a probit or logit regression line represents the change in the proportion of responders per unit change in dose. Toxicological evidence suggested that the slope of a dose–response regression line reflected host enzyme activity [19]. Thus, non-parallel lines might indicate different modes of action of the two toxicants. Parallelism between regression pairs was essential for determining the level at which to compare the effects of two toxicants. Generally, there were three main categories of parallelism: (i) the two regression lines were statistically parallel (e.g., Fairfax vs Pixley; Fig. 2a); (ii) the two regression lines were not statistically parallel but did not cross within the dominant region (20–80%) of the response proportions (e.g., Rotenone vs Deguelin; Fig. 2b); and (iii) the two regression lines crossed around the median lethal dose (e.g., BugRes vs BugLab; Fig. 2c). In the first case, reporting the LD_50_s of the two toxicants and their ratios was sufficient. In the second case, one should report both LD_50_s and LD_90_s (and/or LD_10_s) and their ratios. In the third case, reporting the ratios of LD_10_s, LD_50_s, LD_90_s is meaningless, but the significance of difference between the two slopes should be valid.

## Conclusions

We successfully developed a method to calculate the lethal doses of a toxicant at different significance levels, and compare lethal dose ratios using probit-log(dose) regression by the ML procedure implemented in Microsoft Excel. Lethal doses calculated using this method at different significance levels, as well as lethal dose ratios with their 95% CLs, were identical or close to those calculated using Polo-Plus and SPSS. When judged by whether the 95% CLs of the lethal ratios included 1.0, all methods reached the same conclusions regarding toxicity differences between two samples.

## Additional file

Additional file 1: Calculation of LDs and their ratios.xlsx (344 kb), which requires Microsoft Excel 2010 or higher. It is available via a link: https://figshare.com/s/f94393f752fcc15faea7. (XLSX 1493 kb)

## Abbreviations

95% CLs: 95% confidence limits
LD_50_: Median lethal dose
ML: Maximum likelihood
RMP: Relative median potency
